# Waste Glass Valorization as Raw Material in the Production of Portland Clinker and Cement

**DOI:** 10.3390/ma15207403

**Published:** 2022-10-21

**Authors:** Alina Bădănoiu, Adriana Moanță, Ovidiu Dumitrescu, Adrian Ionuț Nicoară, Roxana Trușcă

**Affiliations:** 1 Department of Science and Engineering of Oxide Materials and Nanomaterials, Faculty of Applied Chemistry and Materials Science, University Politehnica of Bucharest, 011061 Bucharest, Romania; 2 CEPROCIM S.A. Preciziei No. 6, 062203 Bucharest, Romania; 3 National Research Center for Micro and Nanomaterials, Faculty of Applied Chemistry and Materials Science, University Politehnica of Bucharest, 060042 Bucharest, Romania

**Keywords:** soda-lime waste glass, alternative raw material, raw mix, clinker, Portland cement, slag, properties

## Abstract

The paper presents experimental results regarding the synthesis of Portland clinker starting from raw mixes based on two types of clayey precursors, i.e., clay and marl (the most common types of raw materials used in the cement industry), with and without glass waste content. The soda-lime glass waste addition (5.36–5.59 wt %), used to control the silica ratio of the raw mix, improved the raw mix burnability and decreased the calcination temperature (by 20 °C), leading to a decrease in fuel consumption and contributing to the reduction in CO_2_ emissions associated with clinker and cement production. The clinkers obtained by the calcination of raw mixes with glass waste content at 1430 °C with a 30 min plateau had a similar mineralogical composition and microstructure to the clinkers obtained from the reference raw mixes and fulfilled the requirements of the specific standard EN 197-1. The obtained clinkers were used to produce two types of Portland cement, i.e., a unitary cement (CEM I) and a binary blended cement with slag (CEM II/B-S). The main characteristics of these cements, i.e., loss on ignition, insoluble residue, sulfate and chloride contents, as well as the setting time and soundness, meet the conditions stipulated in the EN 197-1 standard. The values of compressive strength, assessed on mortars after 2, 7 and 28 days of curing, allow the classification of all CEM I cements in the 42.5 R class. In the case of CEM II/B-S cements, those obtained from raw mixes with clay can be classified in the 42.5 N class, while those obtained from raw mixes with marl are classified in the 32.5 R class.

## 1. Introduction

Sustainable development is based on economic development, social responsibility and environmental protection. These three pillars are largely dependent on the efficiency of energy consumption and energy resources. Therefore, the production of environmentally friendly building materials with a low carbon footprint is a priority.

Portland cement and concrete are recognized as key construction materials, and this sector is considered indispensable to Europe’s economy [1,2]. Still, the Portland cement industry is considered one of the most energy-intensive industries, which generates up to 8% of CO_2_ anthropogenic emissions [3,4]; moreover, due to its huge output—4.1 billion tons in 2019 [5]—this industry consumes important quantities of raw materials. 

Portland cement is produced by the burning, in a kiln, of a raw mix containing limestone, clay or marl and several additions; the resulting clinker is then intergrinded with gypsum (compulsory) and other admixtures (optional) to obtain cement. The CO_2_ emissions in cement production are mainly generated by the fuel combustion in the kiln and by the decarbonation process of raw materials, especially limestone, which have a high share in the raw mix [6].

Various approaches are considered today to reduce the carbon footprint of this industry. Among these, the use of alternative fuels, increasing the energy efficiency of kilns as well as the production of cements with a low clinker content are solutions already applied at the industrial level [1,4,6,7]. Several types of alternative raw materials are currently used in cement plants for the production of Portland cement; among them, the most used materials are slag (waste from the metallurgical industry) and fly ash (waste from coal-fired power plants). Both can be used as raw materials for Portland clinker production or, more often, as supplementary cementitious materials (SCM) intergrinded with Portland clinker and gypsum [8,9,10,11,12]. 

Waste glass also represents an important environmental issue. The yearly production of glass is 209 million tonnes [13], and due to their brittleness, glass products are easily transformed in waste. The highest share in glass production is represented by container glass, followed by flat glass, domestic glassware, reinforcement glass fibers, special glass and other types of glass [13]. Part of the glass waste (culets) can be recycled in the manufacture of new products, especially glass containers and domestic glassware. With reference to the chemical composition of cullet glass used in the production process, 95% represents soda-lime glass, borosilicate glass and lead glass; the remaining 5% is special purpose glass [14].

The recycling of glass culets in the manufacture of new glass products is a complex process, as the glass wastes should be sorted and cleaned before melting [15]. Therefore, an important amount of waste glass is still landfilled. One possible recycling option for waste glass is its use in the production of building materials such as glass fibers, foam glass, geopolymers, intumescent materials and paints or mortars and concretes based on Portland cement [16,17,18,19,20]. The use of glass waste as an alternative raw material in the production of concrete is the most studied alternative. Crushed glass can be used for the total or partial replacement of cement, sand or coarse aggregate [15,21]. Cementitious concretes containing waste glass exhibit some superior properties as compared with normal Portland concretes, i.e., resistance to acid attack, sulfate resistance, increased resistance to freeze–thaw cycles and shielding properties against harmful gamma rays [16,22]. Nevertheless, the high amount of alkalis present in the composition of waste glass can be deleterious for the mechanical strength of the concrete if alkali–silica reaction (ASR) occurs between the alkalis and reactive silica, potentially present in the used aggregates. This deleterious effect is more intense for coarse glass particles (larger than 1 mm) used for the substitution of natural aggregate in concrete production [16]. However, the ASR reactions can be mitigated using cements containing various mineral admixtures (SCM) such as fly ash, slag or silica fume [23].

Another option to use waste glass is as an alternative raw material in the production of silicate cement [24,25,26]. This approach is less studied due to the complexity of the subject. When cathode ray tube (CRT) glass, with high lead content, was used as raw material for clinker production, the burnability of the raw mix was improved but resulted in Pb losses (0.69% to 42.15% depending on the level of incorporation) [26]. Xie and Xi [24] used scrap glass to replace part of the clayey component in the raw mix used to produce Portland clinker. The raw mixes with waste glass had an improved burnability as compared with conventional raw mix, but the cements obtained from these clinkers had flash setting and poor strength development; the authors explain this by the increase in sodium calcium aluminate (Na_2_O∙8CaO∙3Al_2_O_3_) content in clinkers, favored by the increase in alkali content in the raw mixes with waste glass content. 

This paper presents the synthesis and properties of Portland clinkers and cements with and without waste glass content. We systematically assessed the influence of clayey precursors, i.e., clay or marl, as well as the presence of waste glass (used as alternative raw material to correct the silica content in the raw mixes) on the main properties of clinkers and cement. The obtained clinkers were used to produce two types of Portland cement, i.e., a unitary cement (CEM I) and a binary blended cement with slag (CEM II B-S), for which we assessed the main properties (as required by the specific EN 197-1 standard). The reported results extend the present knowledge regarding the use of waste glass as a raw material for clinker production to the production of cements based on these clinkers with slag addition. The use of slag reduces the alkali content in cement and can contribute to the mitigation of potential alkali–silica reactions in concrete.

## 2. Materials and Methods

### 2.1. Raw Materials

The raw materials used to obtain the studied clinkers and cements were as follows:

- Limestone is the main source of CaO in the raw mix, with a volumetric weight of 2480 kg/m^3^.
- Clay is the main source of SiO_2_ and Al_2_O_3_ in the raw mix, with a volumetric weight of 1870 kg/m^3^.
- Marl is the source of SiO_2_, Al_2_O_3_ and CaO in the raw mix, with a volumetric weight of 2170 kg/m^3^.
- Pyrite cinder—waste from the production of sulfuric acid, with a volumetric weight of 1450 kg/m^3^. Pyrite cinder, is used to correct the Fe_2_O_3_ content of the raw mix.
- Sand—tailing resulting from the preparation of quartz sand; sand is used to correct the SiO_2_ content of the raw mix.
- Mixed colored waste glass (WG) was obtained from a company which collects and processes waste glass from sanitation companies (Bucharest, Romania), municipal waste sorting stations as well as from authorized collectors. WG is used to correct the SiO_2_ content of the raw mix—it replaces sand in some compositions.
- Slag, waste from the metallurgical industry, is used as supplementary cementitious material (SCM) to prepare CEM II B-S cements.

The chemical composition of these materials is presented in Table 1.

**Figure 1:**
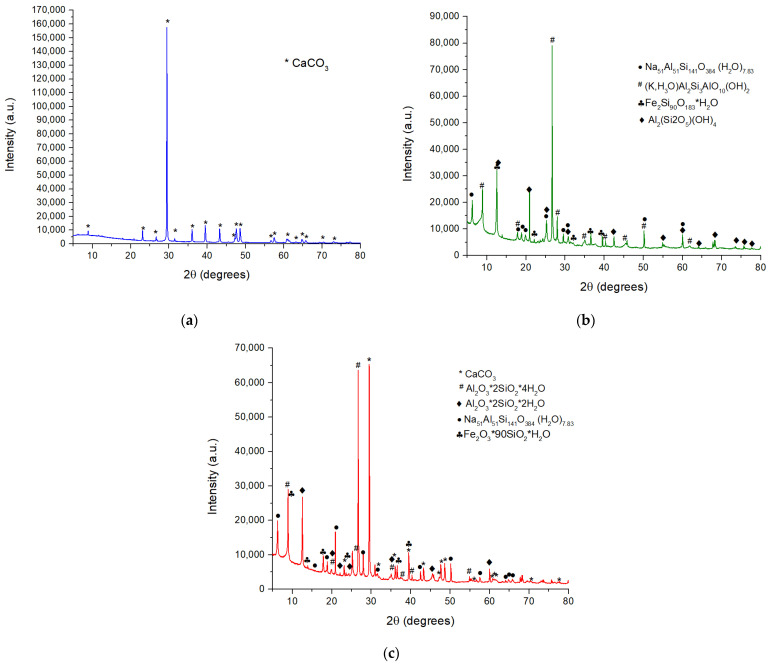
XRD patterns of (**a**) limestone; (**b**) clay; (**c**) marl.

The mineralogical compositions of limestone, clay and marl were assessed by X-ray diffraction (XRD) (Figure 1).

As can be seen form Figure 1a, the main mineralogical compound assessed by XRD in limestone is calcite (ICDD 083-0577). 

The mineralogical composition of clay, assessed by XRD (Figure 1b), is illite (K,H_3_O)Al_2_Si_3_AlO_10_(OH)_2_—ICDD 00-026-0911), kaolinite (Al_2_(Si_2_O_5_)(OH)_4_—ICDD 079-1570), sodium silicate aluminate hydrate (Na_51_Al_51_Si_141_O_384_(H_2_O)_7.83_—ICDD 076-0591) and traces of iron silicate hydrate (Fe_2_Si_90_O_183_H_2_O—ICDD 043-0580).

The main minerals assessed in marl (Figure 1c) are calcite (CaCO_3_—ICDD 083-0578), kaolinite (Al_2_O_3_∙2SiO_2_∙2H_2_O—ICDD 003-0052), halloysite (Al_2_O_3_∙2SiO_2_∙4H_2_O—ICDD 002-0043), sodium silicate aluminate hydrate (Na_51_A_l51_Si_141_O_384_ (H_2_O)_7.83_—ICDD 00-076-0591) and possibly traces of iron silicate hydrate (Fe_2_O_3.90_SiO_2_.H_2_O—ICDD 042-0424). 

Given the fact that waste glass consists mainly of soda-lime glass culets, this material has a high content of silica, lime and alkali (Na_2_O equivalent of about 14%). 

### 2.2. Design and Preparation of Clinkers and Cements 

**Figure 2:**
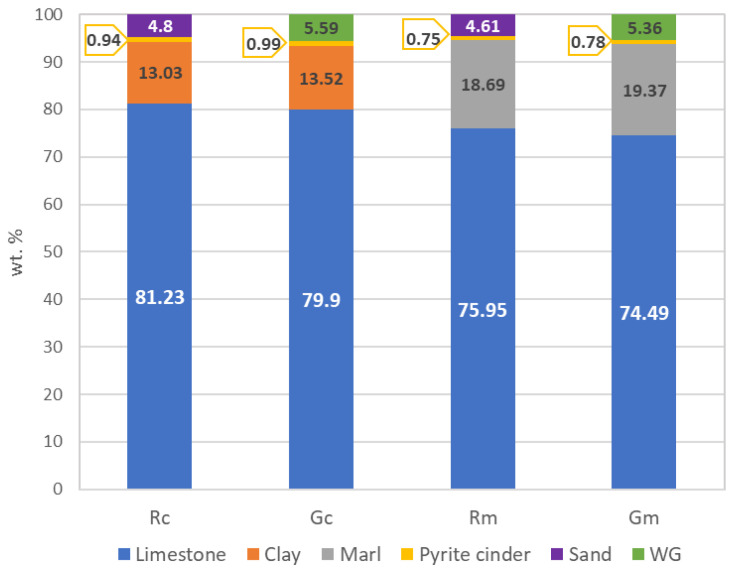
Composition of raw mixes used for clinker and cement manufacture.

To valorize waste glass as a raw material in the manufacture of Portland cement, the recipes of raw mixes were calculated considering the substitution of sand (often used as a corrective addition for the silica ratio) with waste glass (WG). The calculus of the raw material dosage was conducted to obtain clinkers with a lime saturation factor (LSF) of 0.98, a silica ratio (SR) of 2.5 and an alumina ratio (AR) of 1.6. The compositions of raw mixes are presented in Figure 2.

The raw mix Rc is obtained using clay as a silicate aluminate raw material and sand to correct the value of the silica ratio, and in Gc, the sand is replaced by waste glass. Raw mix Rm contains marl and sand (to control the silica ratio), while in Gm, the sand was replaced by waste glass. 

The raw materials were crushed until the grain sizes were below 7 mm, and then milled in a laboratory mill (Ceprocim, Bucharest, Romania) with a rotary drum and discontinuous operation, up to a fineness corresponding to 12% residue on the 90 μm mesh sieve (R_90_ = 12%). The raw mixes were then mixed with water (water to solid ratio of 0.28) and the resulting pastes were shaped into pellets, which were then dried at 100 °C. The thermal treatment was performed in an electric oven, with a rate of 4.83 °C/min at different temperatures and plateaus [9,28]. Free lime was assessed in these clinkers using the ethylene glycol method [29].

In the case of raw mixes with glass waste, in order to optimize the technological thermal treatment parameters, samples of approximately 200 g were submitted to different heat treatments, i.e., maximum temperature 1400 °C/20 min plateau, 1430 °C/30 min plateau and 1450 °C/30 min plateau. The optimal thermal treatment conditions were selected based on the free lime content assessed in the obtained clinkers, i.e., the free lime content should be lower than 2% [30,31,32]. After determining the optimal thermal treatment conditions, the raw mixes (approx. 10 kg) were subjected to the optimal heat treatment and the main characteristics of the clinkers (chemical and mineralogical composition, microstructure) were assessed.

The obtained clinkers with free lime content below 2% were used to prepare two types of Portland cement: CEM I (with 95% clinker and 5% gypsum addition) and CEM II/B-S (65% clinker, 30% granulated blast furnace slag and 5% gypsum addition). 

In the case of cements with additions (CEM II), in order to reduce the alkali content of the final product (cement), we chose the granulated blast furnace slag (waste from metallurgical industry) because the alkali content of this type of material is lower (Na_2_O = 0.54%; K_2_O = 0.49%) than that of other types of supplementary cementitious materials (SCMs) that are frequently used in the cement industry.

The cements were made by the intergrinding, in a laboratory ball mill, of clinker, gypsum (CEM I) and slag (in the case of CEM II/B-S). The grinding was carried out until the cement fineness, expressed as a residue on the 90 μm mesh sieve, was approximately 1%.

### 2.3. Methods

The chemical composition of cements was determined using wet chemistry methods presented in EN 196-2 [33].

The setting time and soundness of the studied cements were assessed on pastes according to the methods presented in the standard EN 196-3 [34], and the mechanical strengths were assessed on mortars prepared and cured according to the method described in the standard EN 196-1 [35].

The microstructure of clinkers was assessed by scanning electron microscopy (SEM) using a QUANTA INSPECT F50 microscope (Thermo Fisher, Eindhoven, The Netherlands), with a field emission gun (FEG) with 1.2 nm resolution and a dispersive energy X-ray spectrometer (EDS) with MnK_α_ resolution of 133 eV. All images were obtained using a secondary electron detector (ETD) at 30 kW acceleration voltage and the usual 3.5 spot.

To determine the mineralogical composition, we used an X-ray diffractometer, PANalytrical Empyrean (PANalytical, Almelo, The Netherlands) in Bragg-Brentano geometry equipped with an X-ray tube with a Cu anode (λCuK_α_ = 1.541874 Å) operated at 45 kV tube voltage and 40 mA tube current. The spectrum was acquired at room temperature (25 °C) in the angle range 2θ of 20–80° with a step size of 0.026°, a time per step of 0.5 s and a revolution speed of 0.5 s. Phase identification and Rietveld quantitative phase analysis were performed using the X′Pert High Score Plus 3.0 software (PANalytical, Almelo, The Netherlands). After the refining process, values were obtained between 1.59% and 2.21% for goodness of fit, between 8.01% and 8.36% for R_expected_ and between 7.48% and 8.89% for R_profile_. All the parameters are below the imposed theoretical limits, which indicates that the applied model is suitable. 

## 3. Results and Discussions

### 3.1. Clinkers Characterization

One of the factors used to assess the quality of clinkers obtained by the calcination of raw mixes is free lime content. Higher values of free lime in clinkers could be detrimental in that they could determine an important volume expansion during the hardening of cement and, consequently, an important reduction in the mechanical strength; these types of cements are classified as unsound [36]. Therefore, it is recommended to keep the free lime content in clinker below 2% [30,31,32,37].

**Figure 3:**
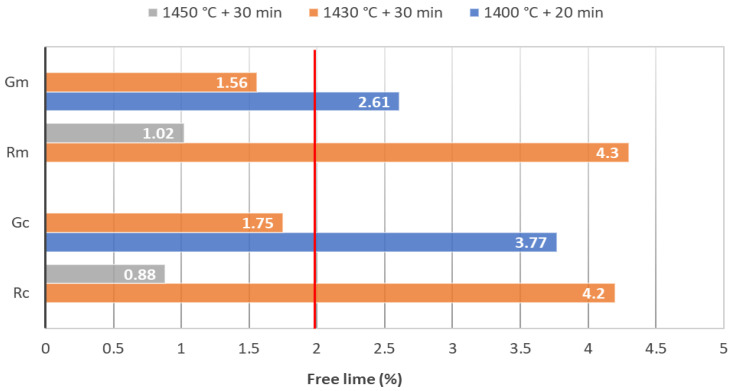
Free lime assessed on the clinkers obtained by calcination at different temperatures and plateaus (red line = maximum free lime content recommended in clinkers).

The free lime content assessed by chemical methods on the clinkers obtained by the calcination of raw mixes based on clay or marl, with sand or waste glass admixtures, are presented in Figure 3.

As expected, the increases in the calcination temperature and plateau duration determine the decrease in free lime content, both in reference clinkers (Rc and Rm) and clinkers resulting from the raw mixes with WG content (Gc and Gm). For the raw mixes with waste glass, the calcination temperature can be reduced by 20 °C, i.e., the clinkers Gc and Gm have a free lime content below 2% when the calcination is performed at 1430 °C with a 30 min plateau. This decrease is explained by the lower melting point of soda-lime glass (below 1000 °C); the addition of this type of waste glass to cement raw mixes decreases the eutectic temperature of these compositions [24].

**Figure 4:**
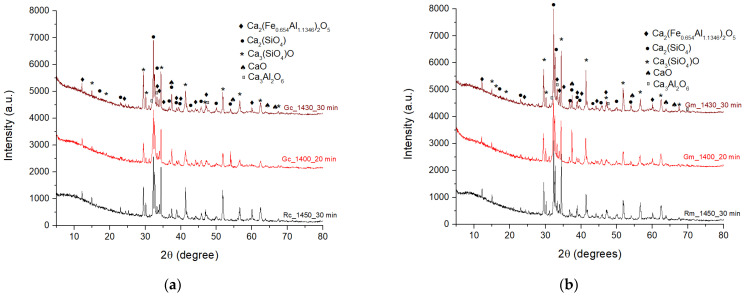
XRD patterns of clinkers obtained by calcination of raw mixes with/without WG content: (**a**) raw mix based on clay; (**b**) raw mix based on marl.

Figure 4 shows the XRD patterns of the Rc and Rm clinkers obtained at 1450 °C with a 30 min plateau and of clinkers with WG (Gc and Gm) calcined at 1400 °C with a 20 min plateau and at 1430 °C with a 30 min plateau. As can be seen, in all clinkers, the crystalline phases detected by XRD are alite (3 CaO·SiO_2_), belite (2 CaO·SiO_2_), calcium iron aluminum oxide Ca_2_(Fe0_,654_Al_1,346_)O_5_ and 3 CaO·Al_2_O_3_, which are the main mineralogical phases usually assessed in Portland clinkers. 

Interestingly, these analyses did not provide information regarding the formation of new phases with alkali content in the clinkers with WG, as suggested in other studies [7,24]. The same mineralogical compounds identified in reference clinkers are present in the XRD patterns of Gc and Gm clinkers. One can assume that alkali ions brought into the system by the waste glass addition are incorporated (solid solutions) in the main mineralogical phases, i.e., alite, belite, calcium iron aluminate and tricalcium aluminate [30].

**Figure 5:**
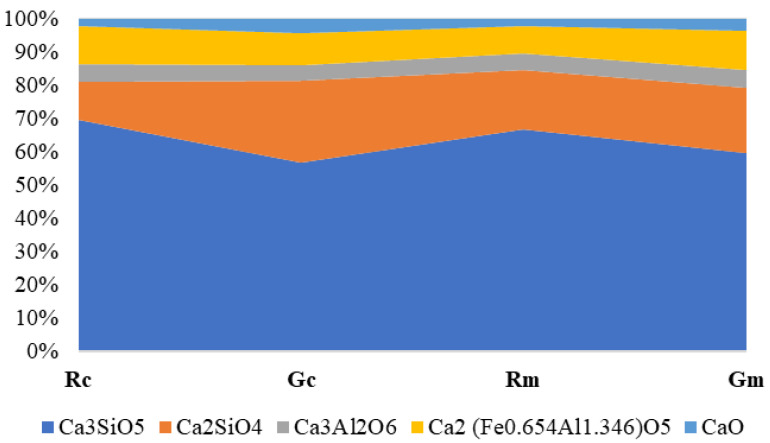
Rietveld quantification of crystalline phases on clinkers obtained by calcination of raw mixes with/without WG content: Rc and Gc—raw mix based on clay; Rm and Gm—raw mix based on marl. Rc and Rm were obtained by thermal treatment at 1450 °C, 30 min; Gc and Gm were obtained by thermal treatment at 1430 °C, 30 min.

Figure 5 presents the phase amounts resulting from the Rietveld refinement performed on the samples in which the WG was introduced into the raw mix based on clay and the raw mix based on marl. One can notice a clear decrease in C_3_S amount in the specimens with waste glass content as compared with the references, correlated with a small increase in C_2_S content.

**Figure 6:**
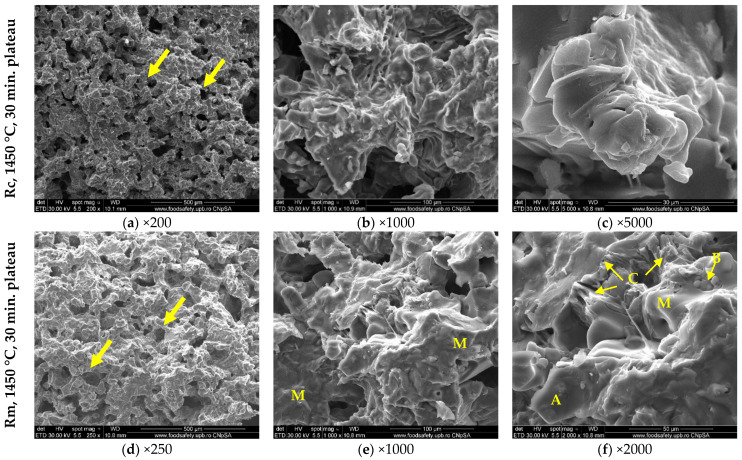
SEM images of Rc and Rm clinkers at different magnifications; A—alite; B—belite; C—calcium ferrite aluminate; M—melt; pores are indicated by arrows.

SEM and EDS analyses performed on clinkers provide supplementary information regarding their microstructure. The SEM analyses of the two reference clinkers with sand and clay or marl as raw materials are presented in Figure 6. 

One can notice the porous structure of clinker particles (some pores are indicated by arrows in Figure 6a,d). At higher magnifications (Figure 6b,c,e,f), one can assess the main specific phases, namely alite (3CaO·SiO_2_), being euhedral particles with clear edges and smooth faces [38,39], and belite (2CaO·SiO_2_), being particles with round shapes [39,40] partially covered with solidified melt (Figure 6f), as well as an interstitial phase (calcium aluminate and calcium ferrite aluminate phases) with a specific elongated “honeycomb” structure [39]. 

**Figure 7:**
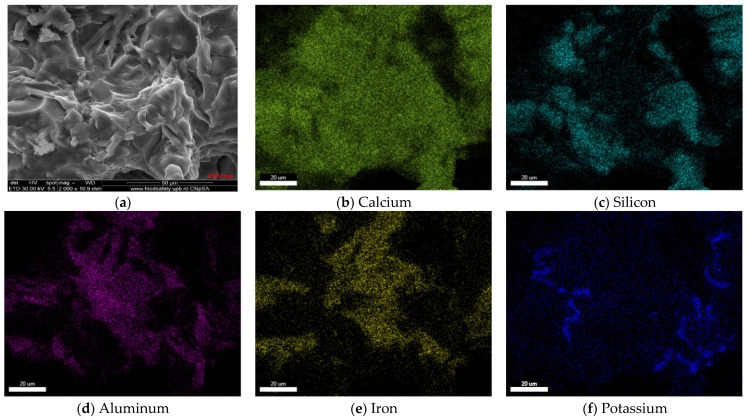
SEM image (**a**) and elemental mapping (**b**–**f**) of Rc clinker.

The EDS elemental maps presented in Figure 7 confirm the wide distribution of calcium in all phases, of silicon in the euhedral and rounded particles, and of aluminum and iron mainly in the interstitial phases. One can also notice in the analyzed micro-area the presence of potassium localized mainly in the interstitial phases. 

**Figure 8:**
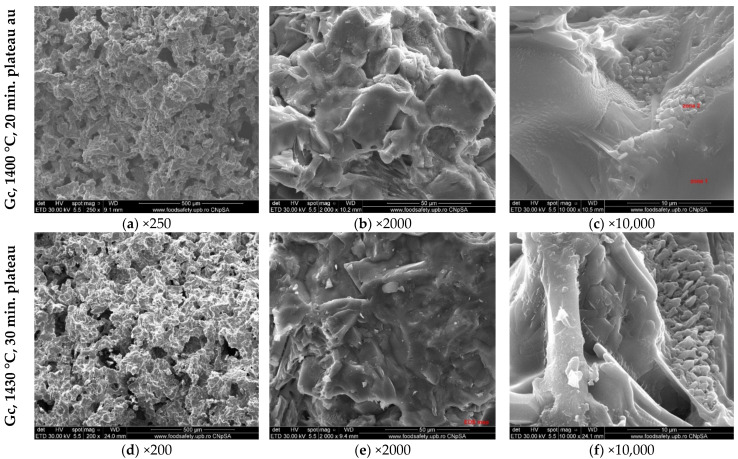
SEM images (different magnifications) of Gc calcined at different temperatures and plateaus (1400 °C + 20 min and 1430 °C + 30 min).

The SEM images of the clinkers obtained by calcination at different temperatures and plateaus of the raw mix with clay and WG are presented in Figure 8.

Both clinkers contain porous particles (Figure 8a,d). At higher magnifications, one can assess the alite and belite particles partially covered with the solidified melt, as well as the interstitial phase (calcium aluminate and calcium aluminate ferrite phases) with the previously described morphologies.

**Figure 9:**
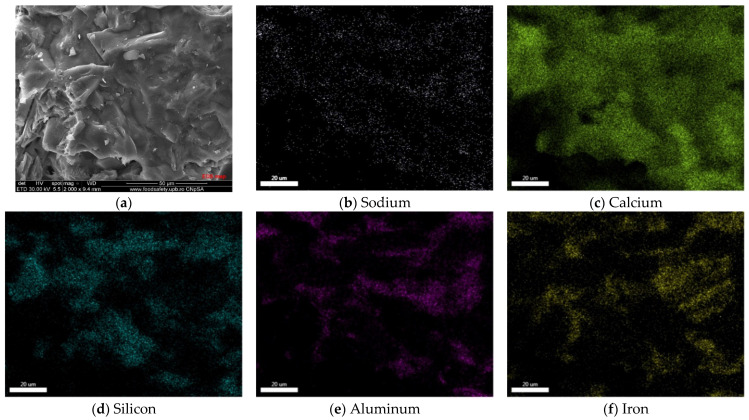
SEM image (**a**) and elemental mapping (**b**–**f**) of Gc clinker.

The elemental mapping of Gc clinker obtained by thermal treatment at 1430 °C with a 30 min plateau (Figure 9) shows the presence of Na (in low amounts), along with Ca, Si, Al and Fe. Sodium seems to be distributed in all phases present in the analyzed micro-area, which could confirm its presence in the silicate and aluminate phases [41].

**Figure 10:**
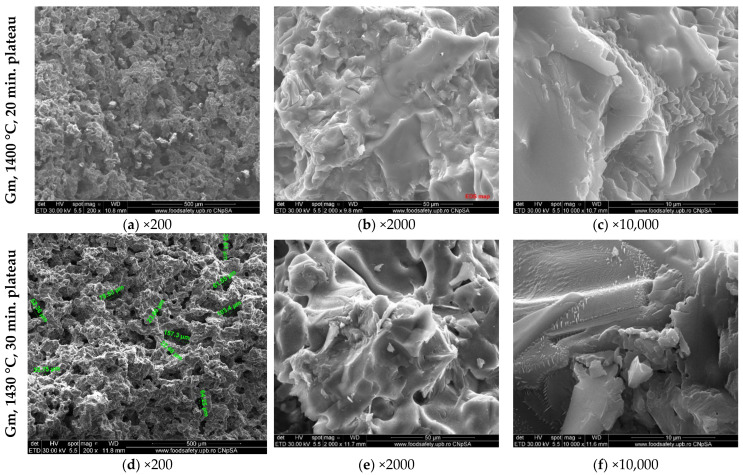
SEM images (different magnifications) of Gm calcined at different temperatures and plateaus (1400 °C + 20 min and 1430 °C + 30 min).

The SEM images of clinkers obtained by the calcination of raw mixes with WG and marl are presented in Figure 10. 

The microstructure is similar to the one assessed for the clinkers with clay, having porous particles; moreover, at higher magnifications, the alite, belite and interstitial phases are identified in the studied micro-area.

Based on the information presented so far, the clinkers with waste glass (Gc and Gm) were obtained by the calcination of raw mixes at 1430 °C with a 30 min plateau, and the reference clinkers (Rc and Rm) were obtained by the calcination of raw mixes at 1450 °C with a 30 min plateau. 

The oxide composition of these clinkers was assessed according to the methods presented in the EN 196-2 standard [33]. Based on these values, LSF, the silica ratio and the alumina ratio, were calculated [41]. The alkali content is expressed as Na_2_O equivalent. The results are presented in Table 2.

As can be seen from Table 2 and Figure 5, all clinkers fulfill the requirements of the EN 197-1 standard [42]:

- The sum of C_3_S and C_2_S (assessed by the Rietveld method) is between 79.20% and 84.80%, which represents more than two-thirds of the mass of clinker.
- The value of the CaO to SiO_2_ ratio is between 3.12 and 3.15 (the standard requires a minimum value of 2).
- MgO content is between 1.6% and 1.99%, much lower than the maximum admissible value of 5%.

The alkali content of the clinkers with WG is higher than those with sand, which was expected given the higher alkali content in the waste glass (see Table 1). 

A high content of alkalis in clinker/cement can negatively influence the properties of the concrete produced with this cement due to potential reactions between the alkalis released by the cement during hydration and the reactive silica which could be present in aggregates (alkali–silica reaction, ASR). Thus, to reduce the effect of potential ASR, careful selection of the aggregates, which should not contain reactive silica minerals, is recommended.

### 3.2. Influence of Waste Glass on the Burnability of Raw Mixes

The burnability of raw mixes is an important technological aspect. It provides information regarding the energy consumption necessary to transform the raw mix into clinker [36,41,43]. The burnability is influenced by the composition of raw materials and raw mixes (LSF, SR and AR values, the content of minor components) as well as the particle size distribution of raw mix (especially the coarse fraction) [41].

The burnability can be assessed based on experimental results (free lime content in clinkers calcinated at different temperatures and plateaus) or can be expressed by calculated indexes, based on oxide and Bogue compositions [27,43,44]. In Table 3 are some of these indexes, their usual (recommended) values and the values calculated for the studied clinkers. 

As can be seen, all indexes calculated for the raw mixes based on clay or marl, with or without WG, are in the ranges specific for clinkers with adequate burnability. 

A higher value of the percentage of liquid phase (*F_liq_*) indicates an easier burning of the raw mix [43]. As can be seen from Table 3, the raw mixes in which sand was substituted with waste glass have higher values for this index, confirming their better burnability, indicated also by the free lime content in clinkers calcined at various temperatures and plateaus. 

Another important parameter for the assessment of raw mix burnability is the heat of reaction, which can be calculated with the formula proposed by Onoda [43], shown in Table 4.

The values presented in Table 4 confirm the positive effect exerted by the replacement of sand with waste glass, i.e., a reduction in the heat of reaction compared to reference clinkers. Since most soda-lime glasses have a melting point below 1000 °C, the addition of this type of waste glass to cement raw mix decreases the eutectic temperature of these compositions [24].

The reduction in the calcination temperature, and implicitly, the reduction in the theoretical consumption of heat necessary for the clinker formation, have positive effects on the environmental impact of clinker and cement production. This allows a decrease in fuel consumption, thus reducing the production costs and at the same time contributing to the reduction in associated CO_2_ emissions.

### 3.3. Cement Properties

The main characteristics assessed for cement, as stipulated in the norm EN 197-1, are:

- Chemical characteristics: loss on ignition (LOI), insoluble residue, sulfate and chloride contents; and
- Physical and mechanical properties: initial setting time, soundness and compressive strengths assessed on mortars after 2, 7 and 28 days of curing.

Given the high alkali content of clinkers with waste glass, the alkali content of cements resulting from the intergrinding of clinkers with 5% gypsum (CEM I) and with 5% gypsum and 30% slag (CEM II/B-S) was also assessed by the method specified in the EN 196-2 standard. 

The chemical characteristics of these cements are presented in Table 5. The cements obtained from clinkers with glass waste (Gc and Gm) meet the chemical conditions stipulated in the EN 197-1 standard, the determined values being below the maximum limits imposed by this norm. 

The use of granulated blast furnace slag as a grinding addition for the preparation of CEM II/B-S cements based on Gc and Gm clinkers (slag partially substituting clinker in cement composition) leads to a decrease in the alkali content, as compared with the corresponding CEM I cements. Furthermore, the alkali content in CEM I cements obtained from Gc and Gm clinkers is smaller as compared with the alkali content of corresponding clinkers (see Table 2), while the CEM II/B-S cements have an alkali content 40% smaller as compared to the corresponding clinkers. Additionally, slag addition to the cement could mitigate the potential harmful alkali–silica reactions [23].

**Figure 11:**
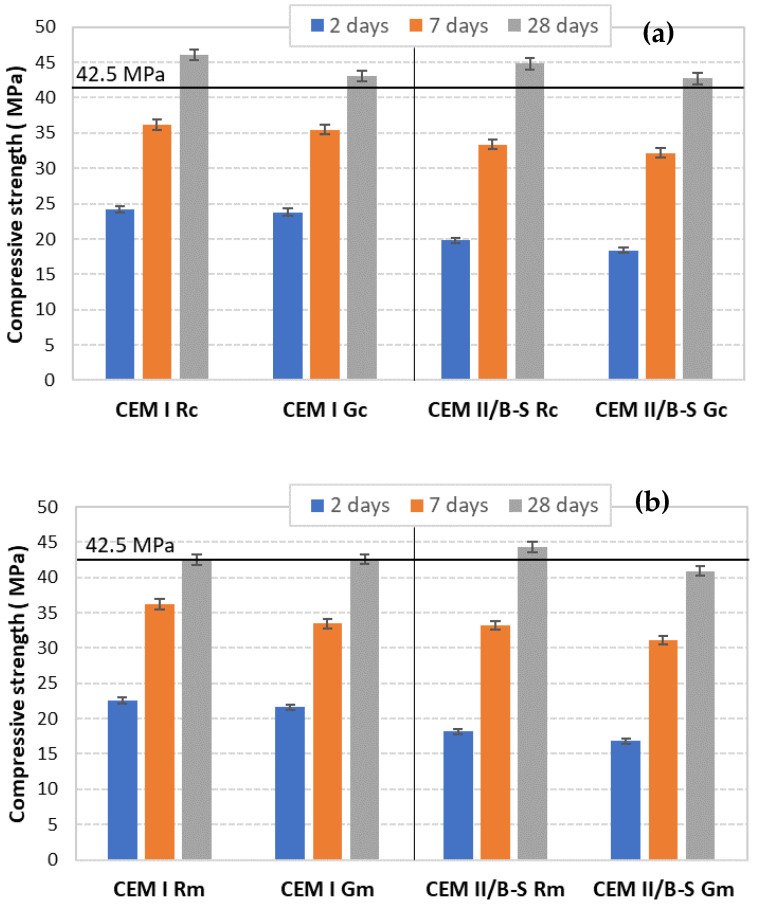
Compressive strengths vs. time for CEM I and CEM II B-S, with sand or WG and based on (**a**) clay; (**b**) marl.

The physical characteristics of cements are presented in Table 6 and the mechanical characteristics are presented in Figure 11.

All cements fulfill the conditions imposed by the EN 197-1 standard for these properties (Table 6). The presence of waste glass in the raw mixes does not significantly affect the initial setting time and soundness of cements. The cements based on marl seem to have a slightly longer initial setting time as compared with those based on clay for both types of cement, CEM I and CEM II/B-S.

The mechanical properties, i.e., the values of compressive strength, presented in Figure 11, correlated with the values of initial setting times (Table 6), leading us to the following conclusions:

- All CEM I can be classified in the 42.5 R class; and
- In the case of CEM II/B-S cements, the one obtained from raw mix with clay (CEM II/B-S Gc) can be classified in the 42.5 N class, while those obtained from raw mixes with marl are included in the 32.5 R class.

The compressive strength values are correlated with the mineralogical composition of the cements assessed by the Rietveld method (Figure 5), i.e., the decrease in the C_3_S amount in the clinkers with waste glass content (Gc and Gm) explains the lower compressive strength recorded for these cements (compared to the references). The partial substitution of clinker with slag determines, as expected, a reduction in compressive strength values, more important for a short hardening time (2 days).

The effects of the use of waste glass, clay and marl as raw materials on the environmental impact (CO_2_ emissions) of clinker and cement production are presented in Table 7. The CO_2_ emissions were calculated based on the theoretical calculation of the greenhouse gas (CO_2_) emissions resulting from the limestone decarbonation process.

From the data presented in Table 7, one can notice a reduction in CO_2_ emissions with 11.32 and 12.70 kg CO_2_/t clinker when waste glass replaces sand in the raw mix composition. The higher values obtained for the raw mixes with marl (as compared to those with clay) are due to the limestone content of marl (see Figure 1). Moreover, the CO_2_ emissions associated with CEM I production decrease by 5% and those associated with CEM II/B-S production decrease by 35%, with reference to the CO_2_ emissions associated with the production of 1 t of clinker with WG.

## 4. Conclusions

The results obtained in this study allow us to draw the following conclusions.

- Soda-lime waste glass can be used as a raw material for Portland clinker and cement production. The complete substitution of sand with waste glass, used for the control of the silica ratio of the raw mix, improves the burnability and decreases the calcination temperature by 20 °C, contributing to the reduction in fuel consumption and thus reducing the CO_2_ emissions associated with clinker and cement production.
- The clinkers obtained by the calcination of raw mixes based on clay or marl and with glass waste addition had a similar mineralogical composition and microstructure to the clinkers obtained from the reference raw mixes (with sand).
- All clinkers obtained in this study (with or without waste glass) fulfill the requirements of the specific EN 197-1 standard, i.e., the sum of C_3_S and C_2_S is between 79.20% and 84.80%, the CaO/SiO_2_ ratio is higher than 2 and the MgO content (1.6–1.99%) is lower than the maximum admissible value of 5%.
- Two types of cements were prepared by the intergrinding of obtained clinkers with gypsum (CEM I—95% clinker and 5% gypsum addition) and with slag (CEM II/B-S—65% clinker, 30% granulated blast furnace slag and 5% gypsum addition). The main chemical characteristics of these cements, i.e., loss on ignition, insoluble residue, sulfate and chloride contents, meet the conditions stipulated in the EN 197-1 standard, the determined values being below the maximum limits imposed by this norm. As expected, the alkali content of the cements obtained from the raw mixes with waste glass is higher as compared with the reference (with sand), but the substitution of clinker with slag in the case of CEM II/B-S leads to a decrease in the alkali content by 20% compared to CEM I.
- The initial setting time and soundness of all studied cements fulfilled the requirements of the specific EN 197-1 standard. The values of compressive strength, assessed on mortars after 2, 7 and 28 days of curing, permit the classification of all CEM I cements in the 42.5 R class; in the case of CEM II/B-S cements, those obtained from raw mixes with clay can be classified in the 42.5 N class, and those obtained from raw mixes with marl are classified in the 32.5 R class.

## Figures and Tables

**Table 1:** Chemical composition (wt %) of raw materials.

Raw Materials	LOI *	SiO_2_	Al_2_O_3_	Fe_2_O_3_	CaO	MgO	SO_3_	Mn_2_O_3_	Na_2_O	K_2_O	Other Comp. **	Na_2_O_echiv_ ***
Limestone	43.42	0.43	0.47	0.74	53.31	0.74	0.29	-	0.33	0.09	0.18	0.39
Clay	8.27	55.60	18.24	7.07	3.76	2.07	0.14	-	1.28	2.94	0.63	3.22
Marl	16.3	40.42	13.04	5.90	17.11	2.36	0.47	-	3.11	3.16	0.18	3.16
Pyrite ash	2.96	9.61	3.07	78.49	1.47	0.57	1.93	-	0.52	0.49	0.89	0.84
Sand	1.58	89.05	4.05	2.06	1.12	0.30	0.22	-	0.33	1.14	0.15	1.08
Waste glass	-	71.30	1.86	0.32	10.13	2.17	-	-	13.49	0.46	0.27	13.79
Slag	0.42	36.93	10.74	1.29	44.34	3.55	0.27	0.85	0.54	0.49	0.58	0.86

* Loss on ignition; ** TiO_2_ and Cr_2_O_3_ for waste glass and TiO_2_ for the other raw materials; *** %Na_2_O_echiv_ = %Na_2_O + 0.658%K_2_O [27].

**Table 2:** Oxide compositions of clinkers.

	Clinker	Rc	Gc	Rm	Gm
Oxide Composition, %					
Loss on ignition (LOI)		0.31	0.19	0.16	0.24
SiO_2_		21.28	20.88	21.13	21.05
Al_2_O_3_		5.56	5.56	5.61	5.45
Fe_2_O_3_		3.37	3.52	3.57	3.23
CaO		66.49	65.76	66.17	65.94
MgO		1.99	1.69	1.99	1.60
SO_3_		0.27	0.27	0.21	0.73
Na_2_O		0.24	1.26	0.30	1.20
K_2_O		0.10	0.45	0.12	0.53
TiO_2_		0.39	0.42	0.74	0.03
Free lime (CaO_f_)		1.06	1.91	1.1	1.8
Na_2_O_echiv._^1^		0.31	1.56	0.38	1.55
Lime saturation factor (LSF) ^2^		0.96	0.96	0.96	0.96
Silica ratio (SR) ^3^		2.38	2.30	2.30	2.43
Alumina ratio (AR) ^4^		1.65	1.58	1.58	1.69

^1^ %Na_2_O_echiv_ = %Na_2_O + 0.658%K_2_O; ^2^ LSF = (CaO − CaO_f_)/(2.8SiO_2_ + 1.1Al_2_O_3_ + 0.7Fe_2_O_3_); ^3^ SR = SiO_2_/(Al_2_O_3_ + Fe_2_O_3_); ^4^ AR = Al_2_O_3_/Fe_2_O_3._

**Table 3:** Raw mix burnability indexes.

Index	Formula	Usual Values	Rc	Gc	Rm	Gm	Source
Burnability index (BI)	$BI=\frac{\%C_{3}S}{\%C_{3}A+\%C_{4}AF}$	2.6 ÷ 4.2	3.24	3.02	3.12	3.18	[25,41,42]
Refractory index (IR)	$IR=\frac{\%C_{3}S+0.6\%C_{3}A-0.61\%C_{4}AF}{1.12\%C_{3}A+1.45\%C_{4}AF}$	1.3 ÷ 3	2.47	2.27	2.35	2.44	[25,41]
Coating tendency (AW)	*AW = %C_3_A + %C_4_AF + 0.2%C_2_S + 2%Fe_2_O_3_*	28 ÷ 30	29.06	29.88	29.91	28.55	[25,41]
Liquid phase (%)	*F_liq_ (for 1450 °C) = 3%Al_2_O_3_ + 2.25%Fe_2_O_3_ + %MgO + %Na_2_O + % K_2_O + %SO_3_*	23 ÷ 28	26.86	28.27	27.48	27.68	[25]

**Table 4:** Heat of reaction (calculated with Onoda formula) for the studied raw mixes.

Clinker	Formula	Rc	Gc	Rm	Gm
Q_Onoda_ (kcal/kg)	Q = 4.8%Al_2_O_3_+ 7.14%CaO + 5.87%MgO-73	440.11	433.13	438.06	433.60

**Table 5:** The main chemical characteristics assessed for the cements obtained from studied clinkers.

Cement	LOI.	SO_3_	Cl^−^	Insoluble Residue	Na_2_O_echiv_.
EN 197-1	≤5% *	≤3.5%	≤0.10%	≤5% *	-
*CEM I*					
CEM I Rc	1.72	2.47	0.002	0.40	0.47
CEM I Gc	2.23	2.38	0.001	0.39	1.11
CEM I Rm	1.86	2.35	0.002	0.39	0.46
CEM I Gm	2.06	2.45	0.002	0.24	1.12
*CEM II/B-S*					
CEM II/B-S Rc	-	1.87	0.004	-	0.52
CEM II/B-S Gc	-	1.76	0.002	-	0.89
CEM II/B-S Rm	-	1.77	0.001	-	0.56
CEM II/B-S Gm	-	1.90	0.002	-	0.91

* This value is imposed only for CEM I.

**Table 6:** Physical characteristics of obtained cements.

Characteristic	EN 197-1	Cement			
		CEM I Rc	CEM I Gc	CEM I Rm	CEM I Gm
Initial setting time, min	≥75	180	180	200	210
Soundness, mm	≤10	0	2	2	2
		**CEM II/B-S Rc**	**CEM II/B-S Gc**	**CEM II/B-S Rm**	**CEM II/B-S Gm**
Initial setting time, min	≥75	190	200	220	210
Soundness, mm	≤10	0	0	0	0

**Table 7:** Calculated CO_2_ emissions associated with clinker and cement production.

CO_2_ Emissions	Rc	Gc	Rm	Gm
CO_2_ emissions due to lime decarbonation (kg/t clinker)	639.35	628.03	641.19	628.49
CO_2_ emissions due to the production of CEM I (kg/t cement)	607.38	596.63	609.13	597.07
CO_2_ emissions due to the production of CEM II/B-S (kg/t cement)	415.58	408.22	416.77	408.52

## Data Availability

Not applicable.
